# Reproducibility of liver iron concentration estimates in MRI through R2* measurement determined by least‐squares curve fitting

**DOI:** 10.1002/acm2.13096

**Published:** 2020-11-18

**Authors:** Andrew M. Headley, Jared V. Grice, David R. Pickens

**Affiliations:** ^1^ Department of Radiology and Radiological Sciences Vanderbilt University Medical Center Nashville TN USA

**Keywords:** HIC, iron overload, LIC, MR relaxometry, R2* relaxometry

## Abstract

Measuring transverse relaxation rate (R2* = 1/T2*) via MRI allows for noninvasive evaluation of multiple clinical parameters, including liver iron concentration (LIC) and fat fraction. Both fat and iron contribute to diffuse liver disease when stored in excess in the liver. This liver damage leads to fibrosis and cirrhosis with an increased risk of developing hepatocellular carcinoma. Liver iron concentration is linearly related to R2* measurements using MRI. A phantom was constructed to assess R2* quantification variability on 1.5 and 3 T MRI systems. Quantification was executed using least‐squares curve fitting techniques. The phantom was created using readily available, low‐cost materials. It contains four vials with R2* values that cover a clinically relevant range (100 to 420 Hz at 1.5 T). Iron content was achieved using ferric chloride solutions contained in glass vials, each affixed in a three‐dimensional (3D)‐printed polylactide (PLA) structure, surrounded by distilled water, all housed in a sealed acrylic cylinder. Multiple phantom stands were also 3D‐printed using PLA for precise orientation of the phantom with respect to the direction of the static magnetic field. Acquisitions at different phantom angles, across multiple MRI systems, and with different pulse sequence parameters were evaluated. The variability between any two R2* measurements, taken in the same vial under these various acquisition conditions, on a 1.5 T MRI system, was <7% for each of the four vials. For 3 T MRI systems, variability was less than 14% in all cases. Variability was <6% for both 1.5 and 3 T acquisitions when unchanged pulse sequence parameters were used. The phantom can be used to mimic a range of clinically relevant levels of R2* relaxation rates, as measured using MRI. These measurements were found to be reproducible relative to the gold‐standard method, liver biopsy, across several different image acquisition conditions.

## 1 INTRODUCTION

Liver iron concentration (LIC) measurement is necessary for evaluation of a variety of iron‐loading disorders including hereditary HFE hemochromatosis, thalassemia, sickle cell anemia, aplastic anemia, and myelodysplasia.^1^, ^2^ Iron overload is a systemic disorder characterized by a high level of iron in the plasma and functional cells and results from excess iron absorption or transfusional iron intake in the liver, endocrine organs, heart, and other organs. High LIC may potentially lead to end‐stage organ damage and increased risk for liver, endocrine, and cardiac complications.^3^ The liver is the main iron storage organ and the first to show iron overload.^4^ For this reason, accurate quantification of LIC is critical in evaluating efficacy of treatment for iron overload.

In MRI, measurement in the liver of effective transverse relaxation rate, R2* = 1/T2*, is directly proportional to LIC and has been shown to accurately estimate LIC when referenced to the gold‐standard measurement technique: chemical analysis of biopsy measurements.^5^ Additionally, reproducibility of MRI‐derived LIC estimates has been shown to be superior to that of biopsy measurements.^6^, ^7^, ^8^, ^9^ Understanding the potential limitations and performance of R2* quantitation for LIC estimation is valuable when implementing this diagnostic tool at large sites.

Previous investigators have used different methods to address related questions. Wood et al. used a single breath‐hold, single echo, gradient echo pulse sequence in vivo where signal intensities from successive images at increasing echo times (TEs) were fit to monoexponential equations with a variable offset. Using transverse relaxation rates to estimate LIC, Wood et al. demonstrated that both R2 and R2* are closely correlated with LIC using data acquired on a 1.5 T MRI system.^5^ The results from the study conducted by Wood et al. address validation of MRI‐based LIC measurements, but not intermachine reproducibility of such measurements. In addition, St. Pierre et al. determined that measured R2 values were found to be highly sensitive and specific for estimating biopsy LICs using R2 relaxometry.^6^ Alústiza et al found that a signal intensity ratio (SIR) method of calculating LIC is reproducible on several different 1.5 T systems.^7^ Therefore, various studies have shown that MR image analysis can be used in conjunction with specified image acquisition techniques to estimate patient LIC. Furthermore, intramachine reproducibility of those measurements has been demonstrated.^5^, ^6^, ^7^ However, evidence of intermachine reproducibility was not extended to R2* estimation techniques. While these studies do not represent an exhaustive search of all published evidence, an extensive literature review suggests further study is warranted concerning intermachine reproducibility of LIC measurements based on R2* quantification.

The goals of this work were to (a) determine whether R2* estimates obtained on different MRI systems are comparable at both 1.5 and 3 T with differing phantom positioning and pulse sequence parameters, (b) create a low‐cost phantom that would evaluate reproducibility of these R2* estimates, and (c) outline a process for optimizing pulse sequences used in clinical R2* quantification.

## 2 MATERIALS AND METHODS

**Fig 1:**
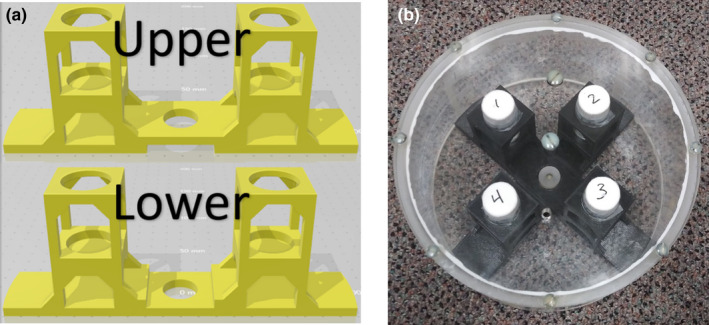
(a) Surface rendering of the computer‐aided design model for the 2‐piece phantom insert and (b) assembled phantom containing glass vials before filling background portion with distilled water. Note that iron concentration in the vials ranges from least in vial 1 through greatest in vial 4.

**Fig 2:**
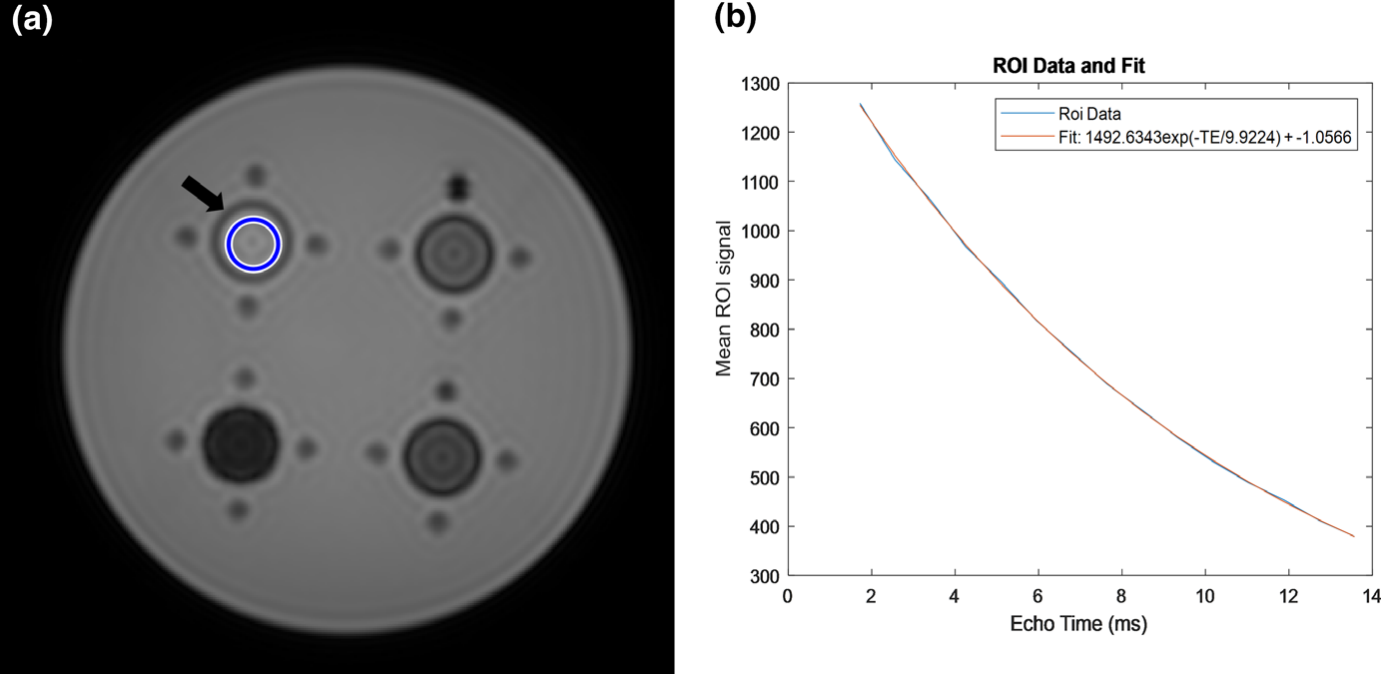
(a) Example region of interest (ROI) (blue circle) placement on a composite image, mid‐vial slice through the phantom (black arrow) and (b) example ROI data are plotted along with the corresponding least‐squares fit. Data for this composite image were acquired using pulse sequence A (see Table 2) on a 1.5 T Philips Achieva. Note equation of the form given in Eq. (1) which can be used to determine R2*.

A two‐piece phantom insert was created via a free, online computer‐aided design (CAD) software called Tinkercad (Autodesk, San Rafael, CA) [Fig. 1(a)] and was 3D‐printed on an entry‐level 3D printer (Creator Pro, FlashForge, City of Industry, CA) using a common, low‐cost polylactic acid (PLA) filament material. The phantom insert was designed to friction‐fit into a pre‐existing acrylic phantom shell and holds four common glass “scintillation” vials [Fig. 1(b)]. The vials are 6.12 cm long, 2.72 cm in diameter, and their walls are 0.23 cm thick. They contain iron concentrations representing a clinical range of R2* values seen in liver iron exams where minimal‐to‐severe iron overload (100‐420 Hz at 1.5 T) is present (Table 1).^10^ Iron concentrations were achieved using known masses of anhydrous ferric chloride (FeCl_3_) dissolved in 0.1 molar aqueous nitric acid (HNO_3_), both measured on a precision balance (ME103TE/00, Mettler Toledo, Columbus, OH). Vials 1‐4 [Fig. 1(b)] were filled with 30 g of HNO_3_ each and approximately 29.4, 41.1, 63.6, and 96.4 mg of FeCl_3_, respectively. The background portion of the phantom was filled with distilled water, minimizing air bubbles. Measurements of R2* were taken in a single mid‐vial slice using a 16 echo, gradient echo pulse sequence. In each case described below, except for the method described in Section 2.C, the signal decay rate, R2*, over 16 sequential images was determined by a least‐squares curve fit of the image data to a monoexponential function with a variable offset

(1) $$S\left(\mathrm{TE}\right)=a\cdot e^{‐\mathrm{TE}\cdot {R2}^{*}}+b$$

where **S** is the average signal in the region of interest (ROI) [Fig. 2(a)] at a given echo time, **TE**, and **a** and **b** are fitting parameters.^11^ The standard deviation of R2* percent difference comparisons from three adjacent slices was used to estimate uncertainty. Nonlinear least‐squares curve fitting and subsequent R2* estimation [Fig. 2(b)] were accomplished using a MATLAB (R2019b, MathWorks, Natick, MA) script which was previously developed at Vanderbilt University. Algorithms used for this fit were Levenberg‐Marquardt and trust‐region reflective. If preset criteria for the fit were not achieved using the Levenberg‐Marquardt algorithm, then the trust‐region reflective algorithm was implemented. Both algorithms are from the Optimization Toolbox of MATLAB.

**Table 1:** Average R2* measured using magnetic resonance imaging.

Field strength (T)	Vial	Average measured R2* (Hz)
1.5	1	103
	2	188
	3	227
	4	420
3	1	144
	2	266
	3	336
	4	616

### 2.A Varied MRI systems, magnetic field strengths, and pulse sequence parameters

Reproducibility of R2* quantification was evaluated using various MRI systems, magnetic field strengths, and pulse sequence parameters. The acquisition protocols used for this part of the study were the same as those used clinically for LIC evaluation at our institution. It is important to note that these pulse sequences were not designed for this experiment, but rather used in current form in order to demonstrate the utility of the phantom as a clinical quality improvement tool. There were two different 16 echo, gradient echo pulse sequences used, acquiring 16 images each, because they were developed by two separate radiology groups at our institution. Since data were collected at both 1.5 and 3 T for the two pulse sequences, they will be referred to as pulse sequences A, B, C, and D. Pulse sequences A and B were used on 1.5 T systems while C and D were used on 3 T MRI systems. Table 2 summarizes the pulse sequence parameters used on both the 1.5 and 3 T MRI scanners. All acquisitions were done with the phantom at ambient scanner room temperature near 20 degrees Celsius but may have varied up to 3 degrees below and above that value.

**Table 2:** Magnetic resonance imaging acquisition parameters for all 1.5 and 3 T scans.

Pulse sequence label	A	B	C	D
Field strength (T)	1.5	1.5	3	3
Number of scanners	3	1	2	1
Minimum TE	0.87	1.501	0.65	1.501
Number of echoes	16	16	16	16
TE spacing (ms)	0.85	2	0.61	2
TR (ms)	400	50	400	50
Flip angle (°)	45	20	45	20
Recon. diameter (cm)	37.5	37.5	35.0	35.0
Recon. matrix	512 × 512	128 × 128	512 × 512	128 × 128
Acq. matrix	124 × 124	88 × 64	124 × 124	88 × 64
rBW (Hz/pixel)	2688	4006	3918	5359
Slice thickness (mm)	7	15	7	15

Pulse sequences B and D are actually subsets of the full clinical pulse sequences, modified to avoid image artifacts that would result in inaccurate R2* quantification. Clinically, for pulse sequences B and D, a 10‐segment pulse sequence was originally used for fine temporal sampling of the signal decay curve. Each of the 10 segments of this pulse sequence consisted of a 16 echo, gradient echo train where echoes were spaced by 2 ms. The first echo of each subsequent segment was shifted by 0.25 ms followed by 2 ms echo spacing. All 160 images were then interleaved in order of increasing echo time to allow for fine sampling, approximately every 0.25 ms, of the R2* relaxation curve.

R2* values for a single vial were estimated across four different 1.5 T Philips MRI scanners (Philips Medical Systems, Amsterdam, Netherlands) using a 16‐element torso coil, where three scanners used pulse sequence A and a fourth scanner used pulse sequence B. Three 1.5 T Philips scanners, two Achieva models and one Ingenia model, employed pulse sequence A. The fourth 1.5 T Philips Intera scanner employed pulse sequence B. Pulse sequence B was used on this scanner because its software revision (R3) did not allow for importing pulse sequence A and pulse sequence B was used clinically on this scanner. The image acquisition and R2* estimation detailed above was repeated on all four vials for each 1.5 T scanner.

R2* values for a single vial were also estimated across three different 3 T Philips scanners, where two scanners (Philips Achieva) used pulse sequence C with a 16‐element torso coil and one scanner (Philips Ingenia Elition X) used pulse sequence D with the 32‐element torso coil supplied with the system. One difference here from the R2* estimation detailed above is that of the 16 echoes collected at 3 T, the earliest echo was removed before the least‐squares curve fit. This was to avoid artifacts present on these 3 T scans. Other than this exception, the same image acquisition and R2* estimation detailed above was repeated on all four vials for each 3 T MRI scanner.

### 2.B Varied phantom orientation

**Fig 3:**
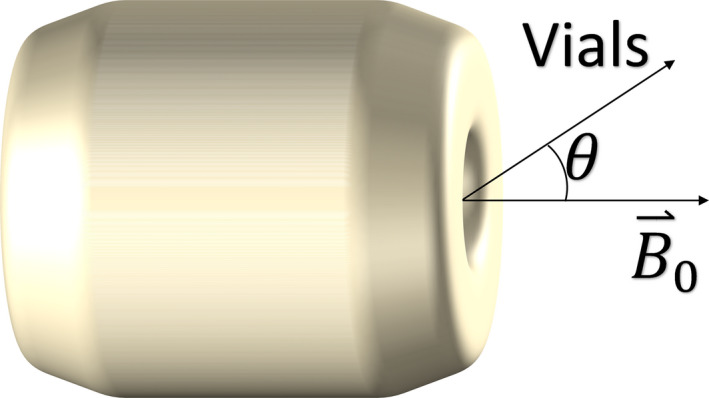
Schematic diagram showing the angular positioning of the vials relative to the static magnetic field of the magnetic resonance imaging system. The “Vials” vector runs parallel to the long axis of the vials.

To verify the position independence of measurements made using the phantom, R2* measurements were compared across different phantom angles on the same MRI system. Support ramps were modeled in CAD and 3D‐printed with PLA to precisely orient the phantom at specified angles (0°, 35.3°, 54.7°, and 90°), relative to the static magnetic field [Fig. 3]. Zero and 90° were chosen as natural angles to set up the phantom, on its side or bottom, respectively. Other angles are intermediate and were randomly chosen. R2* measurements for a single vial were estimated at all four angles, using pulse sequence A on a 1.5 T MRI system, then compared. Slices were obtained obliquely, when appropriate, to create circular cross sections of the phantom in every image. This process was repeated on all four vials.

### 2.C Varied calculation procedures

All calculations, except for those conducted on one 1.5 T Philips Ingenia using a 16‐element torso coil, were carried out using the nonlinear least‐squares curve fitting techniques detailed above. The alternate calculation was executed on the scanner (Philips Ingenia) where a built‐in MR relaxometry software feature was available. This software feature, called mDIXON Quant (Software revision 5.6, Philips Medical Systems, Amsterdam, The Netherlands), is designed and FDA‐approved for liver fat quantification to evaluate hepatic steatosis. This FDA‐approved method is accurate to within 3.5% and reproducible to within 1.4% for fat fraction quantification.^12^ The software feature also allows for calculation of an R2* map, which was used for comparison to the nonlinear least‐squares curve fitting technique developed in‐house.

This 1.5 T MRI scanner was one of the three mentioned Section 2.A using pulse sequence A for intermachine reproducibility evaluation. In addition, this scanner, when acquiring images used for mDIXON Quant analysis, employed an alternate pulse sequence that is recommended for use with this software feature. The possible image outputs for this six‐echo, gradient echo pulse sequence with mDIXON Quant processing are water only, fat only, in‐phase, out‐of‐phase, fat fraction, T2*, and R2*. However, only the R2* map was used for our analysis.

## 3 RESULTS

Using the phantom described earlier, the first parameter that was evaluated in relation to reproducibility of R2* measurements was positioning. On a 1.5 T scanner using unchanged pulse sequence parameters, the variation between R2* measurements for any vial was less than 6% when measurements were taken at various angles between 0° and 90°, as described in Section 2.B. This was found to be true for all four vials containing different iron concentrations [Table 3].

**Table 3:** Percentage difference in R2* values for varied angular positioning.

Angle pair	Vial 1	Vial 2	Vial 3	Vial 4
0° vs 35.3°	2.87%	0.60%	3.90%	1.10%
0° vs 54.7°	3.52%	2.76%	4.32%	0.80%
0° vs 90°	3.59%	1.98%	5.73%	0.48%
35.3° vs 54.7°	0.65%	2.16%	0.42%	1.90%
35.3° vs 90°	0.71%	2.58%	1.83%	0.62%
54.7° vs 90°	0.06%	4.74%	1.41%	1.28%
Average	1.90%	2.47%	2.93%	1.03%

**Fig 4:**
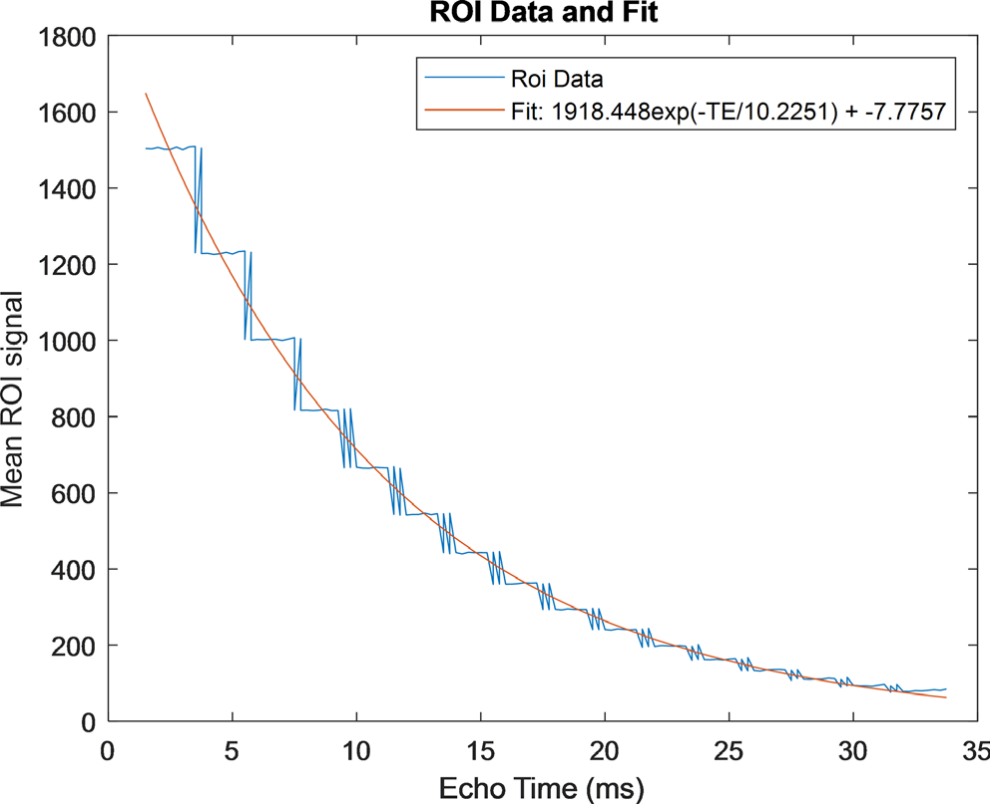
Plot showing stair step artifact in R2* decay curve due to the 10 separate acquisition segments intheclinically used R2* quantification protocol. This region of interest data is from the lowest iron concentration vial.

When the original, 10‐segment versions of pulse sequences B and D were used, image artifacts that would alter R2* quantification were observed. When used clinically, gross patient motion, respiratory motion, and cardiac pulsation are often seen, causing difficulty in R2* estimation. Phantom images acquired with this method revealed a stair step artifact that was visualized in the decay curve [Fig. 4] that had previously been attributed to breathing motion on patient images, but was better isolated in phantom images. This artifact was most likely due to gain adjustments between pulse sequence segments. The artifact was remedied in this study by only using the data obtained from the first segment of the ten‐segment acquisition, leading to 16 echoes rather than 160. None of the R2* values presented in this study were derived from the full 10‐segment clinical protocol, but rather a single subset, as described in Section 2.A.

**Fig 5:**
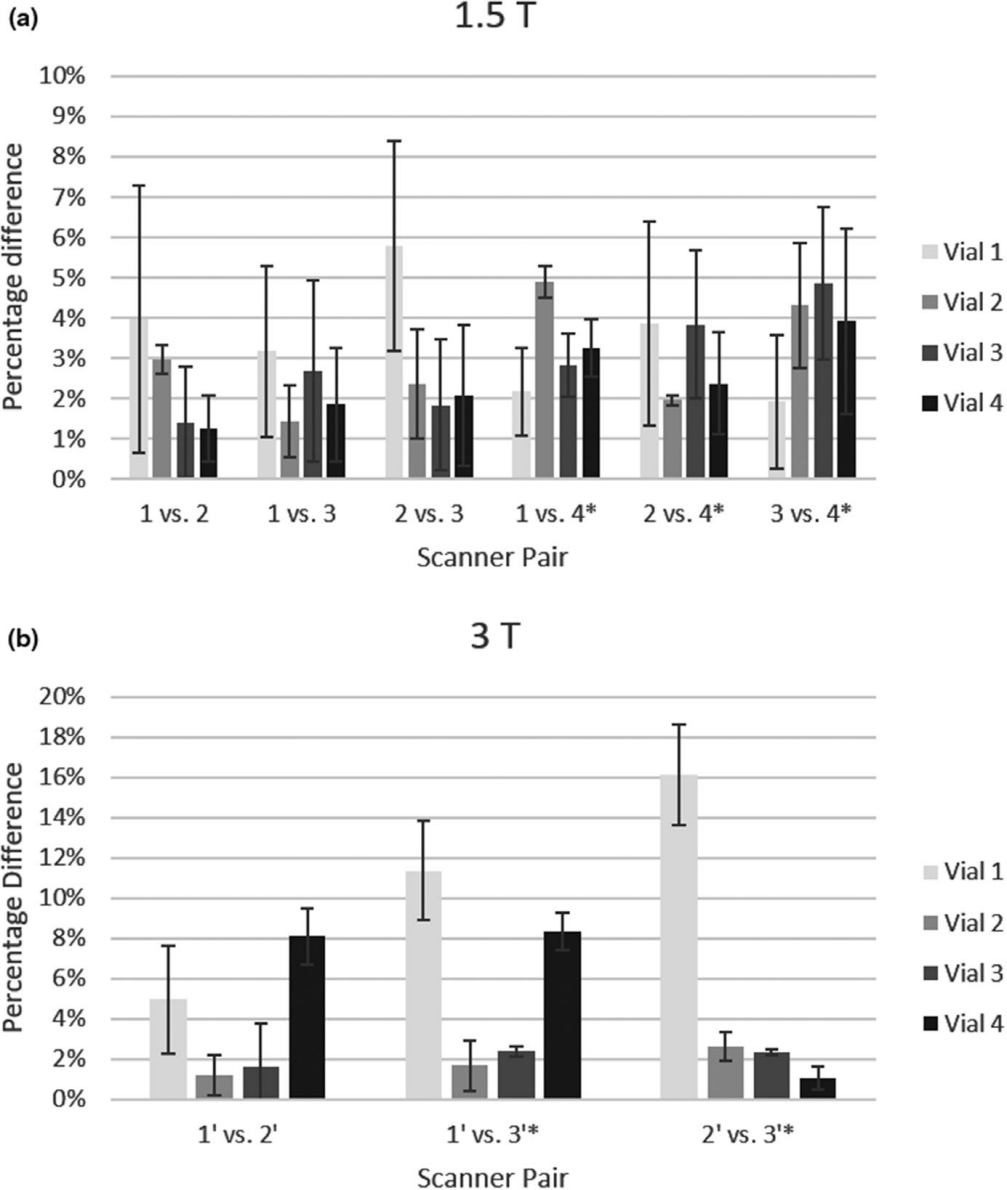
Percentage differences in R2* estimates are shown for each vial for magnetic resonance imaging systems with field strengths of (a) 1.5 T and (b) 3 T. Scanners 4 and 3' (denoted by “*”) used alternate pulse sequences. Note the different scales for percentage difference on each graph.

The process for evaluating intermachine reproducibility of R2* measurements was described in Section 2.A and results are summarized in Fig. 5. The average variation between R2* measurements for any vial was <6% when measurements were taken on various 1.5 T scanners, for the three scanners evaluated using pulse sequence A. When pulse sequence B was used on a different 1.5 T MRI system, variation in R2* measurements increased slightly on average, but was still <6% when all four scanners were compared. The average variation between R2* measurements for any vial was <9% for the two 3 T MRI scanners evaluated using pulse sequence C. When pulse sequence D was used on a third 3 T MRI system, variation in R2* measurements increased considerably, but was <17% when all three scanners were compared.

Finally, measurements comparing R2* quantification for the vendor‐provided software and the in‐house developed method led to R2* variation within 3% forall four vials. These data are summarized in Table 4.

**Table 4:** Percentage difference in R2* values from mDIXON Quant.

Vial 1	Vial 2	Vial 3	Vial 4
1.82%	1.62%	0.14%	2.41%

mDIXON Quant R2* measurements are compared to those obtained on the same MRI scanner with the in‐house developed R2* quantification method.

## 4 DISCUSSION

The purpose of this study was to determine whether quantities derived from R2* quantification, such as LIC, obtained using an MRI system are comparable to those obtained using another MRI system. The phantom created for evaluating reproducibility of R2* estimates was stable over at least five months of scanning. If calibration of a scanner relating measured R2* values to concentrations of elemental iron is desired, then accurate knowledge of the iron concentrations in the vials would be necessary. This could be accomplished using aqueous FeCl_3_ hexahydrate or by employing inductively coupled plasma mass spectrometry where resources allow.^13^ The methods described here posed a problem concerning the quantitative accuracy of weighing anhydrous FeCl_3_. Exposing the FeCl_3_ to air led to immediate absorption of water from the air as evidenced by a steadily increasing mass on the precision balance. Working quickly was the most viable solution given the resource constraints, but accuracy of the prepared iron concentrations corresponding to mg/g of dry liver or aqueous iron could not be expected. However, in‐vivo iron concentrations are based on iron in liver tissue, not aqueous solutions. For this reason, and to achieve the goal of this study, only relative R2* measurements were needed to evaluate reproducibility. Notably, it was important to choose an iron solution where precipitate would not be formed over the period of the study and Alústiza et al.^7^ have shown this type of stability for FeCl_3_ dissolved in HNO_3_.

Phantom features were described in Section 2 and all the features are ideal except the need for a pre‐existing phantom shell, which necessitated designing the phantom insert to friction‐fit inside that existing structure. The phantom shell could be designed and 3D‐printed with the vial holder integrated, but this introduces the challenge of water‐proofing the phantom shell. A potential concern is the degradation of the PLA. Degradation of PLA in normal environmental conditions is complex, but is not likely problematic for a phantom kept in an air‐conditioned environment.^14^ Although we have not seen degradation of our phantom insert after more than a year of submersion in distilled water in an air‐conditioned environment, another phantom insert could easily be 3D‐printed if degradation were visualized. Creating an iron concentration phantom is an essential component of this study, not only providing a reproducible subject for R2* measurements, but also for showing imaging artifacts that are not as easily discerned on clinical image sets.

When considering quantitative imaging using MRI, a clear parameter of concern is the homogeneity of the static magnetic field used for image acquisition. It has been shown that inhomogeneous magnetic fields lead to incorrect evaluation of signal intensity in MR images,^15^ which could ultimately lead to improper characterization of R2*, thus LIC. However, since the magnetic field homogeneity of all MRI scanners at our institution are evaluated annually by qualified medical physicists, as per ACR recommendations, magnetic field inhomogeneity is not expected to account for a substantial portion of the R2* variability measured in this study.

Reproducibility of R2* measurements was evaluated on both 1.5 and 3 T systems, separately. When the same pulse sequence was used on different MRI scanners, less than 6% average variation was seen at 1.5 and 3 T. This is similar to the average variation in R2* measurements seen with varied phantom positioning. When different pulse sequences were introduced, variation in R2* measurements increased slightly on average across the four 1.5 T systems and increased considerably across the three 3 T systems. The levels of variation seen here are slightly beyond the range of 1.4–7% that has been reported previously.^6^, ^15^ is most likely due pulse sequences that will be discussed later in this section. However, variation in R2*, which is linearly related to LIC and is used for iron loading evaluation at our institution, was still less than that which has been reported for multiple needle biopsy measurements in the liver. Variation in needle biopsy results can range from 19% in patients with disease‐free liver to more than 40% for patient with end‐stage liver disease.^6^ For these reasons comparing measurements from multiple MRI systems when evaluating patient LIC indirectly through R2* measurements was deemed acceptable for the MRI systems evaluated.

It was also found that our clinically utilized LIC evaluation protocol and the R2* estimation method used at our institution yielded results that agree to within 3% compared to a technique utilizing the mDIXON Quant pulse sequence and R2* estimation method, which is FDA‐approved for fat fraction estimation via R2* quantification methods.^12^

**Fig 6:**
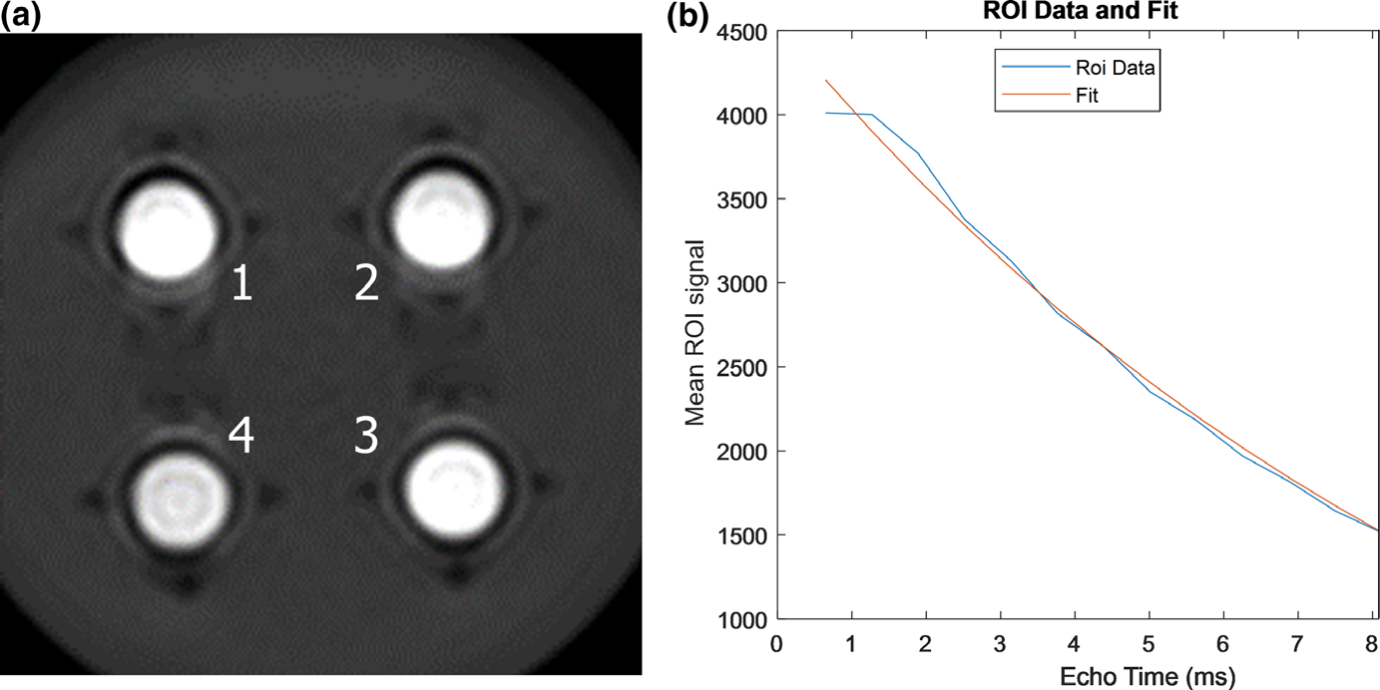
(a) Suspected saturation (bright white signal in vials 1–3) is visualized, especially in vial 1 and (b) signal decay is shown for an region of interest in vial 1. An asymptotic shoulder is exhibited on the plot where the signal, recorded as 12‐bit data, is likely saturated. Data for this composite image were acquired using pulse sequence C (see Table 2) on a 3 T Philips Ingenia Elition X. Note that iron concentration in the vials ranges from least in vial 1 through greatest in vial 4.

As mentioned, it was found that not all pulse sequences should be considered optimal for R2* quantification and these inadequacies may lead to degraded measurement reproducibility. Through the use of clinical R2* quantification pulse sequences for phantom image acquisition, several image artifacts were observed leading to recommendations for protocol modifications. The stair step artifact seen in Fig. 4 was already described in Section 3. Another artifact that was discovered was a shoulder on the R2* decay curve [Fig. 6] from some 3 T acquisitions. For these acquisitions, the increase in signal compared to 1.5 T along with using a pulse sequence designed for a 1.5 T system appears to lead to saturation of the analog‐to‐digital converter [Fig. 6(a)]. This would correspond to a shoulder on a signal decay plot like the one visualized in our data [Fig. 6(b)]. The signal in the asymptotic shoulder is around the maximum pixel value for 12‐bit data (4096) and the artifact is worse in vials with lower iron concentrations, which further supports that the range of signals present in the image had saturated. This led to an altered curve fit and ultimately a decrease in the measured R2* value when the first echo was included in the image set. The artifact was subtle and easily eliminated in most cases by omitting the first echo from the least‐squares curve fits of the R2* data. The artifact was only seen on 3T scanners using sequence C However, this artifact could indicate that some pulse sequences and scanners may be incompatible with this phantom and technique. Generally removing the first echo was a viable solution to this artifact, but more work is necessary to discover all limits of the phantom’s compatibility with varied sequences and hardware.

**Fig 7:**
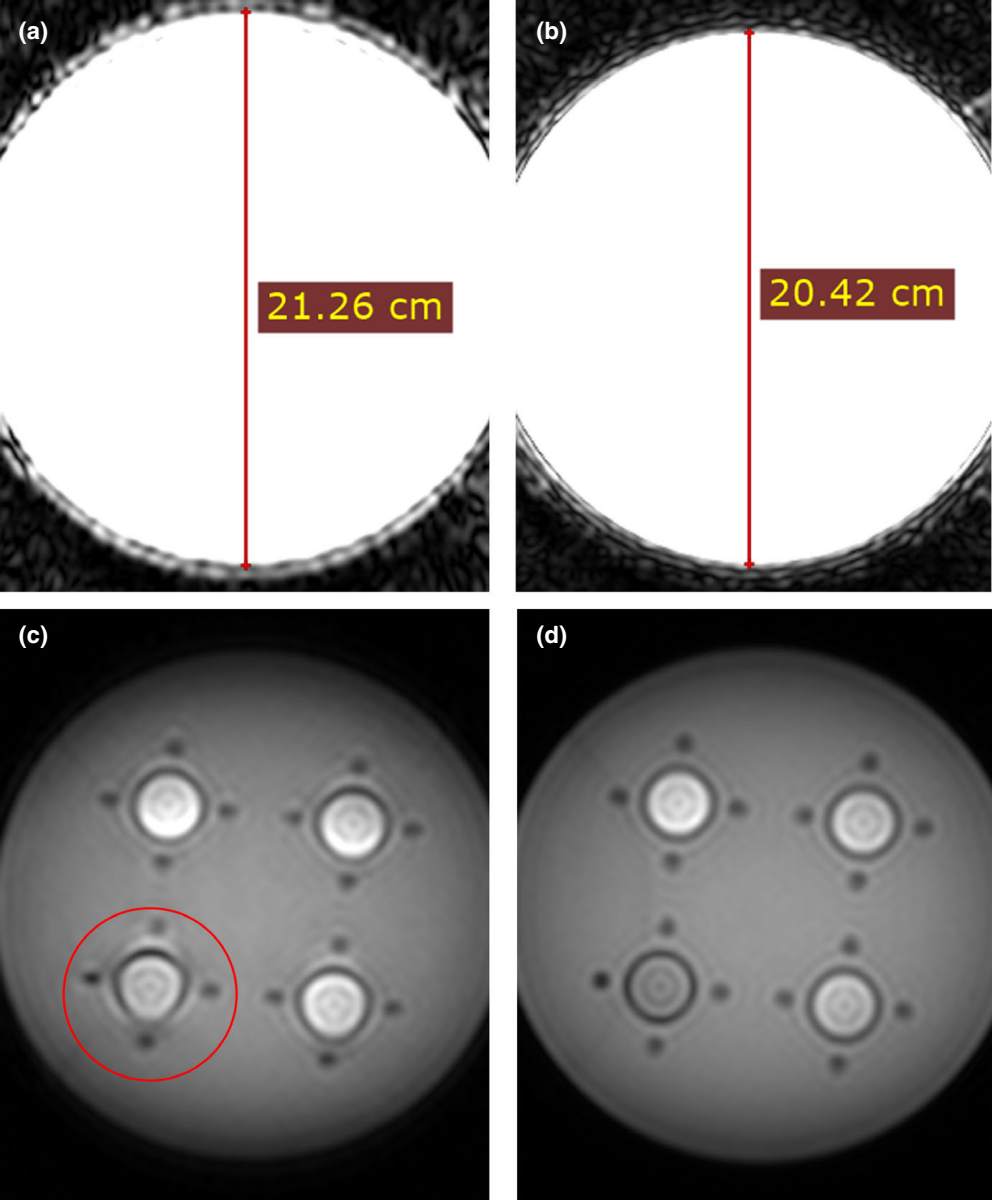
Images of the first (a) and second (b) echo images at the same scale show distortion of the image in the first echo. The window width and level on the images highlights the phantom edges. The glass vials also exhibit distortion (red circle) in the first echo image (c) which is not present in the subsequent images (d). These images were acquired using pulse sequence C (see Table 2) on a 3 T Philips Achieva. The phantom measures 20.4 cm in all images after the first echo.

Spatial distortion of the first echo in the frequency encoding direction was also noted [Fig. 7] in both original pulse sequences regardless of minimum echo time. This artifact could be corrected by ensuring that partial‐echo k‐space techniques are not being used. However, partial‐echo k‐space acquisition was used in this study because the clinical protocols were used without alteration when possible. Omission of the first echo during image analysis for the 3 T data was used to address this artifact.

The final artifact that impacted R2* estimation was additional spatial distortions of the vials in the images perceived as a “jiggling” of the vials in the images when viewed in sequence. This artifact results from unknown phase errors introduced by bipolar multi‐echo readouts. The artifact can be eliminated by applying flyback gradients to the acquisition of k‐space data, allowing for monopolar readouts.^17^ However, a method for correcting this artifact was discovered late in the data acquisition process, so acquisitions were carried out without flyback gradients.

Additionally, though voxel size can be a concern in other quantitative MR methods, such as BOLD MRI, partial volume averaging was not a problem in this study since the vial length is much larger than the slice thickness used and the vial diameter is much larger than the largest pixel size used.

All the described image artifacts are believed to lead to inaccuracy in R2* estimates and increased variability in intermachine measurements. This conclusion stems from the variable severity in appearance of each artifact from scanner to scanner. Each of the artifacts, including susceptibility artifacts, were exacerbated by the use of a higher field strength 3 T MRI system compared to artifacts found in images acquired at 1.5 T. It was also suggested by consulted MR scientists that gradient spoiling should be used and proper shimming should be ensured when dynamic shimming is available. Since most of the artifact reduction techniques described were discovered late in the data collection process, the only technique applied was removal of the first data point from each 3 T data set.

Further work should be done to evaluate intermachine R2* reproducibility when the suggested artifact reduction techniques have been applied to the pulse sequences. It is recommended that a phantom study be done at any institution using MRI for R2* quantification to evaluate pulse sequence‐related artifacts and reproducibility of measurements across various scanners of the same magnetic field strength, since our results only apply to the MRI systems we tested with the pulse sequences used in this study.

## 5 CONCLUSION

Measurements of R2* were insensitive to overall subject positioning. Estimation of R2* was found to be relatively reproducible across different MRI systems, with different pulse sequence parameters, and using different R2* calculation methods. In all cases evaluated, the variation in measured R2*, which is linearly related to LIC, was small compared to multiple liver biopsy evaluations of LIC.^5^, ^6^ Additionally, it was determined that reproducibility of R2* estimates may be improved by implementing several modifications to the pulse sequences evaluated for this study. These include avoiding concatenating data from multiple acquisitions into a single R2* decay curve, avoiding partial‐echo k‐space acquisition, applying flyback gradients, and continuing to use 1.5 T magnetic field strength for R2* evaluation. While this may not be a comprehensive list of pulse sequence parameters, it is important that the same quantification techniques be used every time patient LIC is evaluated.^18^ Assessment of R2* reproducibility should be carried out at every institution that uses R2* quantification for patient management to verify these results for the fleet of MRI systems available.

## CONFLICT OF INTEREST

No conflict of interest.
